# Real-world evaluation of nivolumab in patients with non-nasopharyngeal recurrent or metastatic head and neck cancer: a retrospective multi-center study by the Turkish Oncology Group (TOG)

**DOI:** 10.1007/s00405-024-08744-4

**Published:** 2024-05-25

**Authors:** Arif Akyildiz, Deniz Can Guven, Baris Koksal, Beliz Bahar Karaoglan, Derya Kivrak, Rashad Ismayilov, Firat Aslan, Osman Sutcuoglu, Ozan Yazici, Ahmet Kadioglu, Ozkan Alan, Nargiz Majidova, Mert Erciyestepe, Erkan Ozcan, Goncagul Akdag, Hakan Taban, Ali Osman Kaya, Murad Guliyev, Nilgun Yildirim, Teoman Sakalar, Dogan Yazilitas, Caglar Unal, Sercan On, Sedat Biter, Nebi Serkan Demirci, Filiz Cay Senler, Yasemin Kemal, Omer Diker Halil, Ibrahim Gullu, Sercan Aksoy

**Affiliations:** 1 Department of Medical Oncology, Hacettepe University Cancer Institute, Ankara, Turkey; 2 Department of Medical Oncology, Ankara University Faculty of Medicine, Ankara, Turkey; 3 Department of Medical Oncology, University of Health Sciences, Antalya Training and Research Hospital, Antalya, Turkey; 4 Department of Internal Medicine, Hacettepe University Medical School, Ankara, Turkey; 5 Department of Medical Oncology, Ankara Medical Park Hospital, Ankara, Turkey; 6 Department of Medical Oncology, Gazi University Faculty of Medicine, Ankara, Turkey; 7 Department of Medical Oncology, Dr. Abdurrahman Yurtaslan Oncology Training and Research Hospital, Ankara, Turkey; 8 Department of Medical Oncology, Koç University, Istanbul, Turkey; 9 Department of Medical Oncology, Marmara University, Pendik Training and Research Hospital, Istanbul, Turkey; 10 Department of Medical Oncology, Prof. Dr. Cemil Tascioglu City Hospital, Istanbul, Turkey; 11 Department of Medical Oncology, Trakya University Faculty of Medicine, Edirne, Turkey; 12 Department of Medical Oncology, University of Health Science, Kartal Dr. Lutfi Kirdar City Hospital, Istanbul, Turkey; 13 Department of Medical Oncology, Samsun Training and Research Hospital, Samsun, Turkey; 14 Department of Medical Oncology, Biruni University Faculty of Medicine, Istanbul, Turkey; 15 Department of Medical Oncology, Istanbul University, Cerrahpasa School of Medicine, Istanbul, Turkey; 16 Department of Medical Oncology, Firat University Faculty of Medicine, Elazig, Turkey; 17 Department of Medical Oncology, Necip Fazil City Hospital, Kahramanmaras, Turkey; 18 Department of Medical Oncology, Ankara Etlik City Hospital, Ankara, Turkey; 19 Department of Medical Oncology, Gayrettepe Florence Nightingale Hospital, Istanbul, Turkey; 20 Department of Oncology, Ege University Faculty of Medicine, Izmir, Turkey; 21 Department of Medical Oncology, Cukurova University Faculty of Medicine, Adana, Turkey; 22 Department of Medical Oncology, Altinbas University Faculty of Medicine, Samsun, Turkey; 23 Department of Medical Oncology, Near East University Hospital, Nicosia, Cyprus

**Keywords:** Head and neck cancer, Nivolumab

## Abstract

**Objectives:**

Head and neck cancers (HNCs) represent a significant global health concern due to high morbidity and mortality rates. Despite therapeutic advances, the prognosis for advanced or recurrent cases remains challenging. Nivolumab obtained approval for recurrent or metastatic HNC based on the Phase III CheckMate 141 trial. This study aimed to evaluate the real-world outcomes of nivolumab in patients with non-nasopharyngeal HNC.

**Design:**

In this multicenter retrospective study, we analyzed 124 patients with recurrent or metastatic non-nasopharyngeal HNC who received nivolumab in the second-line setting and beyond. Data were collected from 20 different cancer centers across Turkey. The effectiveness and safety of the treatment and survival outcomes were evaluated.

**Results:**

Nivolumab exhibited favorable clinical responses, yielding an objective response rate of 29.9% and a disease control rate of 55.7%. Safety assessments revealed a generally well-tolerated profile, with no instances of treatment discontinuation or mortality due to side effects. Survival analysis disclosed a median overall survival (OS) of 11.8 (95% CI 8.4–15.2) months. Multivariate analysis revealed that ECOG-PS ≥ 1 (HR: 1.64, p = 0.045), laryngeal location (HR: 0.531, p = 0.024), and neutrophil-to-lymphocyte ratio > 3.5 (HR: 1.97, p = 0.007) were independent predictors of OS.

**Conclusions:**

Nivolumab is an effective and safe treatment option for patients with recurrent or metastatic non-nasopharyngeal HNC in real-world settings. Further studies are needed on factors affecting response to treatment and survival outcomes.

## Introduction

Head and neck cancer (HNC) is the sixth most prevalent cancer worldwide and the eighth most common cause of cancer-related death [1–3]. It encompasses a diverse group of malignancies arising from the oral cavity, pharynx, larynx, nasal cavity, or paranasal sinuses. Despite therapeutic advances, including surgery, radiation therapy, or chemotherapy, the prognosis for patients with advanced or recurrent disease remains challenging [4–6]. Twenty to forty percent of patients with head and neck squamous cell carcinoma (HNSCC) will experience local/regional recurrence or metastasis following primary treatment [5, 7]. Therefore, there is a critical need for novel therapeutic approaches to improve outcomes in this population.

Recently, immunotherapy has emerged as a promising modality in various malignancies, including HNC [8]. Nivolumab, a programmed cell death protein 1 (PD-1) inhibitor, has demonstrated significant clinical activity and improved survival outcomes in patients with advanced HNSCC [8, 9]. These studies have highlighted the potential of nivolumab to improve survival outcomes and provide durable responses in patients with advanced or recurrent HNC. However, the real-world effectiveness and safety profile of nivolumab in a multicenter setting, specifically in the second-line and subsequent treatment, remain to be fully elucidated [10–12].

In this study, we aimed to present the results of a multicenter retrospective analysis focusing on Turkish patients with HNC who received nivolumab as a second-line or subsequent therapy. By assessing real-world data from multiple centers, we assessed the clinical effectiveness and safety profile of nivolumab and survival outcomes in this population.

## Patients and methods

### Patient selection

The study included ≥ 18-years-old patients with histologically confirmed non-nasopharyngeal recurrent or metastatic, platinum-refractory HNC from 20 different cancer centers across Turkey. The study encompassed patients who had previously received at least one course of systemic therapy in the metastatic context or those who had experienced relapse following local treatments. The eligibility criterion was receiving at least 3 cycles of nivolumab as second or next-line therapy. The demographic characteristics of the patients, Eastern Cooperative Oncology Group-performance status (ECOG-PS) and neutrophil–lymphocyte ratio (NLR) at the start of nivolumab therapy, primary tumor locations, histological diagnosis, p16 or programmed death-ligand 1 (PD-L1) positivity, and previous treatments were examined retrospectively. Nivolumab was administered as an intravenous perfusion over 30 min, at intervals of 14 or 21 days. Patients were radiologically evaluated every two to four cycles by ^18^F-fluorodeoxyglucose positron emission tomography/computerised tomography or magnetic resonance imaging. The Response Evaluation Criteria in Solid Tumors (RECIST) was employed to assess the treatment response. History and physical examination and blood work including hemoglobin, white blood cells, platelets, liver function tests, creatinine, thyroid stimulating hormone, and cortisol were done at each participating center shortly before each cycle. Toxicity was categorized using the Common Toxicity Criteria for Adverse Events (CTCAE, version 4.03). This study was conducted in accordance with ethical guidelines and was approved by the respective institutional review boards of all participating centers.

### Study outcomes

The primary outcomes of the study were the efficacy and safety of nivolumab. Efficacy endpoints included overall response rate (ORR, sum of complete and partial response rates) and disease control rate (DCR, sum of complete and partial response and stable disease rates). Safety assessments focused on the incidence and severity of adverse events. The secondary objective of the study was to reveal survival outcomes and prognostic factors.

### Statistical analysis

Descriptive statistics were presented as frequency (percent), mean ± standard deviation (SD), or median (minimum–maximum). The χ^2^ and Exact tests were used to compare the proportions in different categorical groups. The time from the onset of nivolumab to death from any cause was defined as overall survival (OS); to radiological progression or death was defined as progression-free survival (PFS). Survival estimates were calculated with the Kaplan–Meier method. The impact of prognostic variables on OS was investigated through univariate Cox regression analysis. Multivariate analysis was conducted to examine the independent effects of factors that demonstrated significance in univariate analyses and had complete data for all patients. A 5% type-I error level (p < 0.05) was used to infer statistical significance.

## Results

### Patient characteristics

The mean age of 124 patients (73.4% men) was 59.4 ± 13.7 (range, 20–88) years, and 66 (53.2%) patients were over 60 years of age. Smoking history was present in 76 of the 98 patients whose information was available. ECOG-PS was 0 in 63 (50.8%) patients, 1 in 46 (37.1%) patients, and 2 in 15 (12.1%) patients. The most common primary tumor site was the larynx (34.7%), and the others were the oral cavity (29%), hypopharynx (18.5%), oropharynx (8.1%), paranasal sinus (5.6%), and others (4%), respectively. The histological subtype was squamous cell carcinoma in 97.6% of the patients. Among the examined patients, the p16 positivity rate was 23.6% (13/55), and the PD-L1 positivity rate was 51.5% (17/33). Distant metastasis sites were lung (46%), bone (25.8%), soft tissue (21.8%), liver (16.1%), brain (3.2%) and other organs (8.1%), respectively. Treatments before nivolumab included chemoradiation (70.2%), surgery (50%), adjuvant (38.7%), and induction (24.2%) therapies. The number of treatment lines before nivolumab was 1 in 54 (43.5%) patients, 2 in 35 (28.2%) patients, and ≥ 3 in 35 (28.2%) patients. The median dose of nivolumab was 200 (100–240) mg, and the median number of cycles was 6 (3–80). The cycle interval was 14 days and 21 days in 104 (83.9%) and 20 (16.1%) patients, respectively. Concurrent radiotherapy with nivolumab was administered to 18 (14.5%) patients. At the beginning of nivolumab treatment, the median NLR was 3.6 (0.8–19.2) (Table 1).

**Table 1:** Patient characteristics (total of 124 patients)

Characteristics	Frequency, *n (%)*
Age, mean ± SD, years	59.4 ± 13.7
> 60 years	66 (53.2)
Men	91 (73.4)
Smoking *(out of 98 patients)*	76 (77.6)
ECOG-PS	
0	63 (50.8)
1	46 (37.1)
2	15 (12.1)
Primary tumor location	
Larynx	43 (34.7)
Oral cavity	36 (29)
Hypopharynx	23 (18.5)
Oropharynx	10 (8.1)
Paranasal sinus	7 (5.6)
Others	5 (4)
Histological diagnosis	
Squamous cell carcinoma	121 (97.6)
Others	3 (2.4)
p16 positivity *(out of 55 patients)*	13 (23.6)
PD-L1 positivity *(out of 33 patients)*	17 (51.5)
Metastasis sites	
Lung	57 (46)
Bone	32 (25.8)
Soft tissue	27 (21.8)
Liver	20 (16.1)
Brain	4 (3.2)
Others	10 (8.1)
Previous treatments	
Chemoradiation	87 (70.2)
Surgery	62 (50)
Adjuvant	48 (38.7)
Induction	30 (24.2)
No. of previous lines	
1	54 (43.5)
2	35 (28.2)
≥ 3	35 (28.2)
Nivolumab dose, median (min–max), mg	200 (100–240)
Number of cycles, median (min–max)	6 (3–80)
Cycle interval	
14 days	104 (83.9)
21 days	20 (16.1)
Concomitant radiotherapy	18 (14.5)
NLR, median (min–max)	3.6 (0.8–19.2)

*ECOG-PS* Eastern Cooperative Oncology Group-Performance Status, *NLR* neutrophil-to-lymphocyte ratio, *SD* standard deviation

### Efficacy and safety

**Fig. 1:**
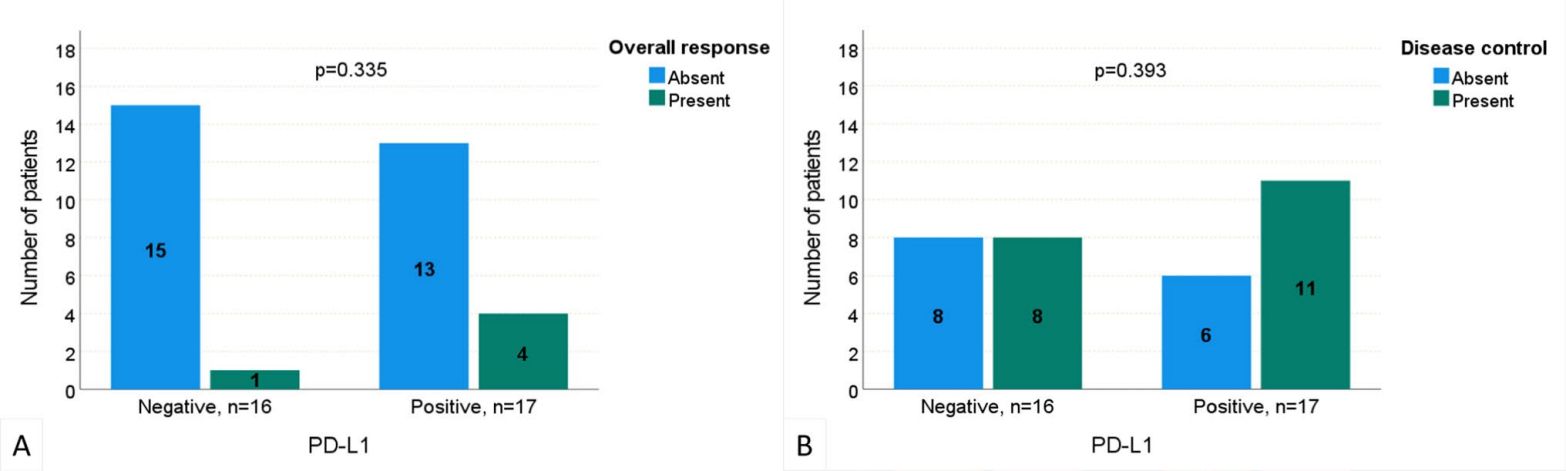
Comparison of PD-L1 negative and positive patients according to the presence of overall response (**a**) and disease control (**b**)

The best responses to nivolumab treatment were evaluated in the study. Complete and partial responses were obtained in 8 (6.5%) and 29 (23.4%) patients, respectively. The disease remained stable in 32 (25.8%) patients, but progressed in 55 (44.4%) patients. Herewith, the ORR was 29.9% and DCR was 55.7%. Neither ORR nor DCR was associated with PD-L1 positivity (p = 0.335 and 0.393, respectively, Fig. 1). Nivolumab was generally well tolerated. There was no treatment discontinuation or mortality due to side effects. Grade 1–2 side effects were encountered in a total of 10 (8.1%) patients (5 hypothyroidism, 2 hypophysitis, 1 pneumonitis, 1 hepatotoxicity, and 1 malaise).

### Survival analysis

**Fig. 2:**
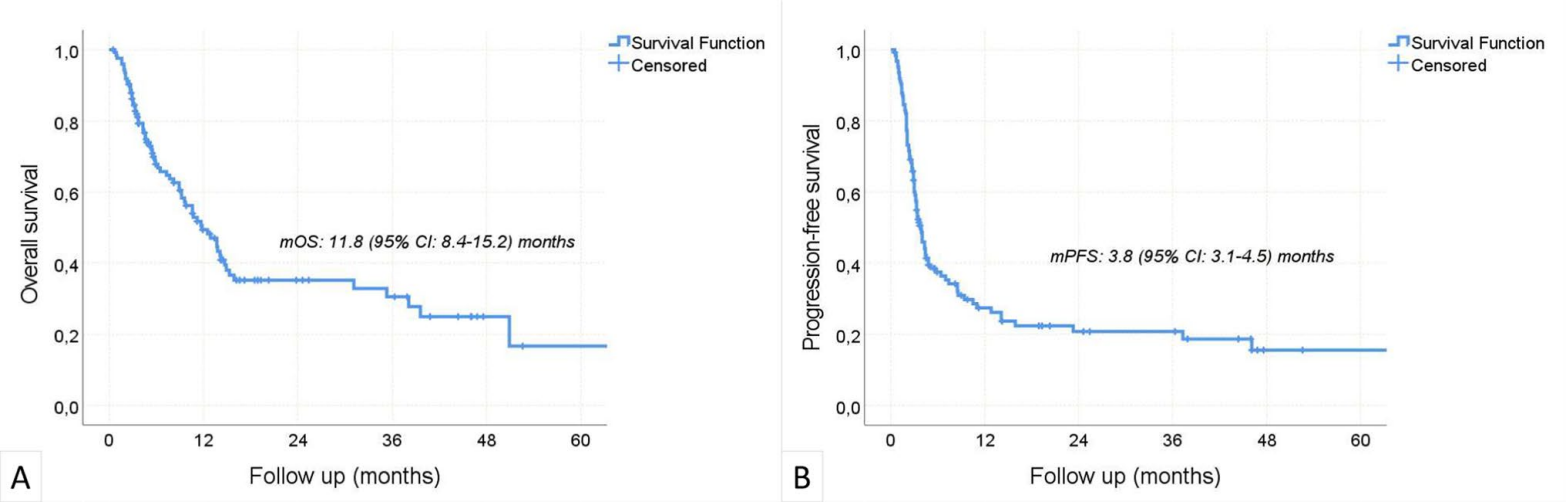
Overall survival (**a**) and progression-free survival (**b**)—Kaplan Meier curves

**Fig. 3:**
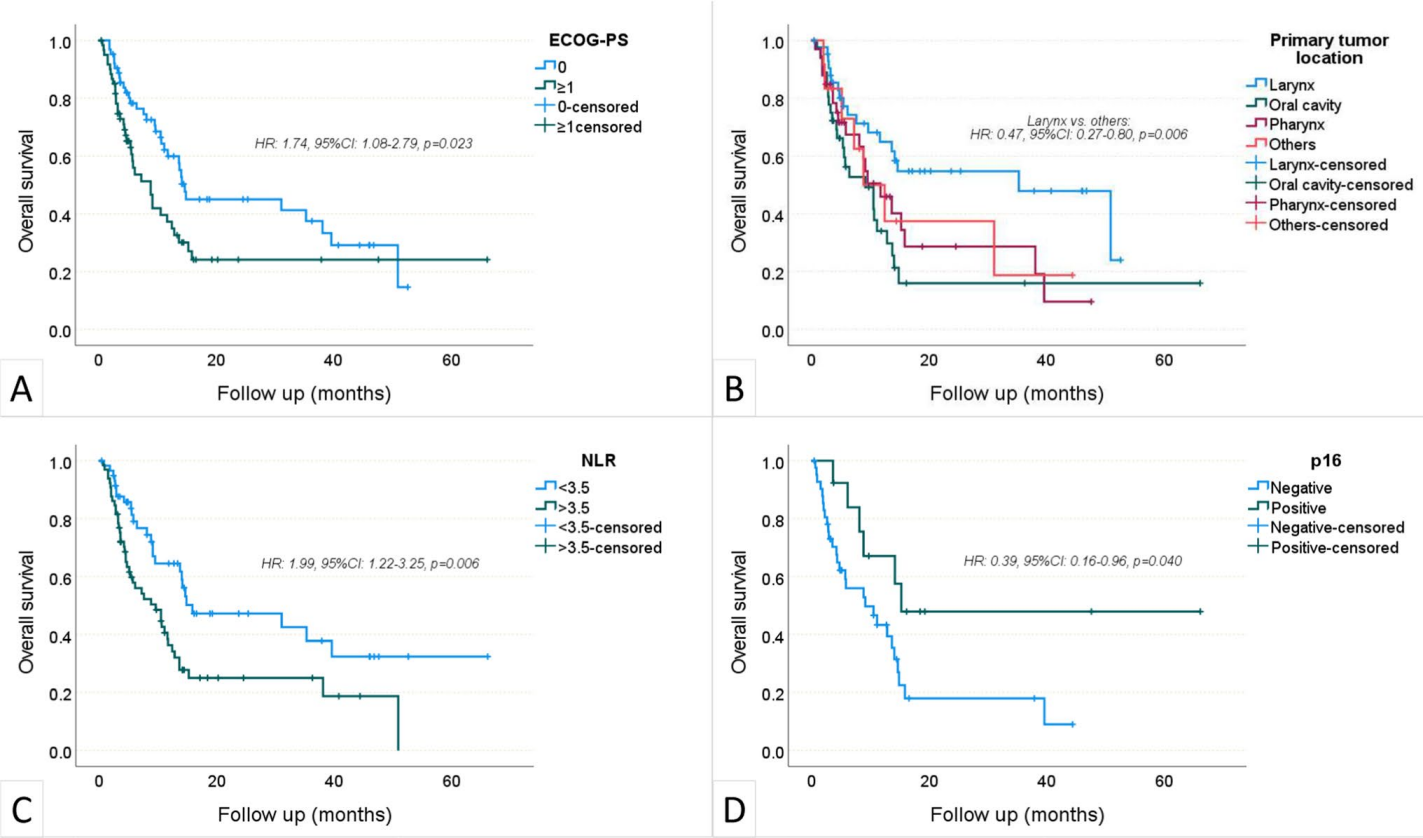
Associations between ECOG-PS (**a**), primary tumor location (**b**), NLR (**c**), and p16 positivity (**d**) with overall survival—Kaplan–Meier curves

During a median follow-up period of 7.5 (0.5–66.1) months, the disease progressed in 89 patients and 70 patients died. The median OS was 11.8 (95% CI 8.4–15.2) months, and the median PFS was 3.8 (95% CI 3.1–4.5) months. The 1-year OS and PFS rates were 49.4% (95%CI 39.6–59.2) and 27.4% (95%CI 18.8–36), respectively (Fig. 2). In univariate analyses, age > 60, male gender, smoking, PD-L1 positivity, metastasis sites, previous treatments, number of previous treatment lines, nivolumab dose, cycle interval, and concurrent chemotherapy were not found to be associated with OS (Table 2). ECOG-PS ≥ 1 (p = 0.023) and NLR > 3.5 (p = 0.006) negatively affected OS, while laryngeal location (p = 0.006) and p16 positivity (p = 0.04) were associated with increased OS. The independent effects of factors found to be significant in univariate OS analyzes were examined by multivariate model. P16 positivity was excluded due to an insufficient number of data (55 of 124 patients). Finally, multivariate analysis revealed that ECOG-PS ≥ 1 (HR: 1.64, p = 0.045), laryngeal location of the primary tumor (HR: 0.53, p = 0.024), and NLR > 3.5 (HR: 1.97, p = 0.007) were independent predictors of OS (Fig. 3 a, b, c). The impact of p16 positivity on OS was investigated using a distinct model comprising 55 patients who underwent p16 testing (Table 3). The analysis unveiled that p16 positivity significantly enhanced overall survival, irrespective of ECOG-PS, primary tumor location, and NLR (HR: 0.39, %95CI 0.16–0.98, p = 0.045, Fig. 3 d).

**Table 2:** Univariate and multivariate Cox regression analysis for overall survival

Risk factors	Univariate Analysis			Multivariate Analysis		
	HR	95% CI	p	HR	95% CI	p
Age > 60 years	1.34	0.83–2.14	0.230	–	–	–
Male sex	0.96	0.57–1.61	0.887	–	–	–
Smoking^£^	1.06	0.55–2.03	0.858	–	–	–
ECOG-PS ≥ 1	1.74	1.08–2.79	**0.023**	1.64	1.01–2.65	**0.045**
Primary tumor location						
Larynx	0.47	0.27–0.80	**0.006**	0.53	0.31–0.92	**0.024**
Oral cavity	1.74	1.06–2.86	**0.028**	–	–	–
Pharynx	1.23	0.73–2.08	0.426	–	–	–
Others	1.12	0.51–2.46	0.769	–	–	–
p16 positivity*	0.39	0.16–0.96	**0.040**	–	–	–
PD-L1 positivity^&^	0.72	0.27–1.91	0.724	–	–	–
Metastasis sites				–	–	–
Lung	0.77	0.48–1.23	0.273			
Bone	0.73	0.40–1.31	0.285			
Soft tissue	1.10	0.61–1.98	0.758			
Liver	1.24	0.66–2.31	0.504			
Brain	0.36	0.05–2.58	0.307			
Others	0.61	0.24–1.51	0.285			
Previous treatments				–	–	–
Chemoradiation	1.08	0.64–1.84	0.767			
Surgery	1.26	0.79–2.03	0.326			
Adjuvant	1.17	0.72–1.88	0.522			
Induction	0.96	0.57–1.62	0.885			
No. of previous lines						
2 (as per 1)	0.84	0.48–1.48	0.557	–	–	–
≥ 3 (as per 1)	0.78	0.44–1.39	0.393			
Nivolumab dose ≥ 200 mg	0.90	0.55–1.47	0.681	–	–	–
Cycle interval of 21 days	0.99	0.92–1.09	0.975	–	–	–
Concomitant RT	0.74	0.40–1.37	0.343	–	–	–
NLR > 3.5	1.99	1.22–3.25	**0.006**	1.97	1.20–3.22	**0.007**

*CI* confidence interval, *ECOG-PS* Eastern Cooperative Oncology Group-Performance Status, *HR* hazard ratio, *NLR* neutrophil-to-lymphocyte ratio, *RT* radiotherapy

Out of ^£^98, ^*^55, and ^&^33 patients

Bold text indicates statistical significance at p < 0.05

**Table 3:** Multivariate Cox regression analysis to assess overall survival in a cohort of 55 patients who underwent p16 testing

Risk factors	HR	95% CI	p
ECOG-PS ≥ 1	1.68	0.82–3.47	0.158
Laryngeal location of the primary tumor	0.53	0.20–1.41	0.201
p16 positivity	0.39	0.16–0.98	**0.045**
NLR > 3.5	1.76	0.85–3.68	0.131

*CI* confidence interval, *ECOG-PS* Eastern Cooperative Oncology Group-Performance Status, *HR* hazard ratio, *NLR* neutrophil-to-lymphocyte ratio

Bold text indicates statistical significance at p < 0.05

## Discussion

In this study, we shared our clinical experience with nivolumab treatment in patients with recurrent or metastatic non-nasopharyngeal HNC in Turkey. Although there are studies in the literature involving patients with nasopharyngeal cancer and HNC receiving immunotherapy [13–15], this study focused exclusively on non-nasopharyngeal cancer due to its distinct differences in terms of epidemiology, histology, natural progression, and response to treatment when compared to other HNCs.

Our cohort comprised 124 patients, with a notable male predominance of 73.4%, consistent with the higher incidence of HNC in males [16]. The mean age of 59.4 years, with 53.2% of patients over 60 years old, reflects the wide age distribution of HNC diagnoses, emphasizing the importance of tailored treatments across different age groups [17]. Evaluating the efficacy of nivolumab in this cohort, we observed encouraging responses, with complete and partial responses achieved in 6.5% and 23.4% of patients, respectively. Stable disease was noted in 25.8% of patients. As a result, the ORR to nivolumab treatment reached 29.9%, while the DCR was 55.7%, underscoring the potential clinical benefit of nivolumab in recurrent or metastatic HNC. These results were notably consistent with the Checkmate-141 trial [18]. Furthermore, the effectiveness and safety of nivolumab in this context have been corroborated by various retrospective studies conducted with diverse real-world data, demonstrating an ORR ranging from 13.5% to 29.6% and a median PFS spanning from 3.7 to 6.4 months [19–21]. The longer PFS observed in our study may be attributed to the less frequent performance of response evaluations in patients, as opposed to the rigorous monitoring in clinical trials.

Survival analysis provided valuable prognostic insights, with a median OS of 11.8 months. The 1-year OS and PFS rates were 49.4% and 27.4%, respectively, highlighting the need for more effective treatments in recurrent or metastatic HNC. In a Japanese study involving patients with recurrent or metastatic HNSCC, it was observed to be relatively high, with a median OS of 74.1 weeks [22]. In a different study, the median OS was found to be 9.2 months [14]. In the Korean study, the median OS was found to be 11.8 months [23], which is longer than the Checkmate 141 study (7.5 months). The variation in reported survival rates between studies conducted in Asian and Western societies highlights the potential differences in disease progression between these regions. Nevertheless, there is a paucity of data in the literature regarding patients receiving nivolumab for HNC in the Middle Eastern population. However, as deduced from our study and the literature, nivolumab offers a superior survival benefit compared to chemotherapy in patients with recurrent or metastatic HNC. Based on the data in this study and the existing literature, it is apparent that PFS is not a reliable indicator in immunotherapy studies, whereas OS holds greater significance.

Nivolumab demonstrated an overall favorable safety profile in our study. Notably, there were no instances of treatment discontinuation or mortality attributed to side effects. Grade 1–2 side effects were encountered in a total of 8.1% of patients, comprising various manageable adverse events. In the Checkmate 141 clinical trial, grade 3 or 4 adverse events were reported in 13.1% of the nivolumab group. Two treatment-related deaths were reported in the nivolumab group, while treatment discontinuation due to severe side effects has not been documented [18]. The lower reported side effect profile in our study may be attributed to potential underdiagnosis in retrospective real-life data, as opposed to controlled clinical trials. However, in our study, no instances of treatment discontinuation or treatment-related deaths resulting from serious immune-related side effects were observed. These data indicate that nivolumab is a safe option for patients with HNC.

Univariate analyses identified factors associated with OS, including ECOG-PS ≥ 1 and NLR > 3.5, both negatively affecting OS. Conversely, laryngeal location and p16 positivity were associated with increased OS. Multivariate analysis confirmed ECOG-PS ≥ 1, laryngeal location, and NLR > 3.5 as independent predictors of OS in the entire cohort. Furthermore, in a subgroup analysis of 55 patients who underwent p16 testing, p16 positivity emerged as the sole independent factor. The Checkmate-141 study demonstrated a more significant survival benefit in the subgroup with PD-L1 expression ≥ 1 (HR: 0.55, 95% CI 0.36–0.83) compared to the PD-L1 < 1 subgroup (HR: 0.89, 95% CI 0.54–1.45) [18]. In our cohort, PD-L1 status was assessed in a limited subset of 33 patients, revealing positivity in 51.5% (17/33) of cases. Univariate analysis indicated that PD-L1 positivity did not have an impact on survival. Given the incomplete patient examination and the small sample size, different results might have been obtained in a clinical trial. The significance of PD-L1 expression, particularly at the 1% threshold, remains debated. Although higher PD-L1 levels were linked to improved survival in the KEYNOTE-012 study, later research suggests that even patients with lower PD-L1 expression levels may benefit from immune checkpoint inhibitors [24]. Therefore, further studies involving a larger patient population are necessary to validate these findings. On the other hand, p16 positivity was identified as a factor positively influencing survival. Consistently, in Checkmate 141 study, the median OS among patients with p16-positive tumors was 9.1 months, whereas it was 7.5 months in the nivolumab group for patients with p16-negative tumors (HR: 0.73, 95% CI 0.42–1.25, p = 0.55) [18]. It is possible that the dynamics of the NLR, reflecting the antitumor immune response during immunotherapy, are directly linked to peripheral treatments and subsequent positive clinical outcomes [25]. NLR serves as a broad-spectrum indicator and can act as a surrogate for the balance between tumor-mediated therapy and antitumor immunity, influencing both cancer progression and overall systemic therapy efficacy [26]. High pretreatment NLR has been correlated with adverse outcomes and may also be associated with inadequate response to immunotherapy [26–28]. Similarly, in our study, an NLR score > 3.5 was identified as one of the factors significantly negatively impacting survival (p = 0.007).

Our study shares several limitations common to retrospective studies. It employed an observational design, lacking a control group. Given that data were collected by individual physicians during routine clinical practice, patient medical records may not consistently offer complete, comparable information and may include measurement errors. Clinical response assessment relied on physician evaluation, which was not standardized within this real-world study. Additionally, to ensure a sufficient sample size, we included healthcare centers with substantial HNC patient populations, which might introduce selection bias into our study population.

## Conclusion

In conclusion, our multicenter retrospective study in Turkish patients with recurrent or metastatic non-nasopharyngeal HNC receiving nivolumab treatment provides valuable real-world insights. The study underlines the potential of nivolumab as a therapeutic option and the importance of personalized treatment approaches. Despite study limitations, including its retrospective nature and sample size, our findings contribute to the growing body of evidence in support of nivolumab in recurrent or metastatic HNC. Further research endeavors are warranted to validate these observations and advance the care and outcomes of patients with HNC.

## Data Availability

The data supporting this study’s findings are available from the corresponding author, upon reasonable request.

## Abbreviations

DCR: Disease control rate
ECOG-PS: Eastern Cooperative Oncology Group-performance status
HNC: Head and neck cancer
HNSCC: Head and neck squamous cell carcinoma
HR: Hazard ratio
NLR: Neutrophil–lymphocyte ratio
ORR: Overall response rate
OS: Overall survival
PD-1: Programmed cell death protein 1
PD-L1: Programmed death-ligand 1
PFS: Progression-free survival
SD: Standard deviation

## References

[1] Bray F, Ferlay J, Soerjomataram I, Siegel RL, Torre LA, Jemal A (2018) Global cancer statistics 2018: GLOBOCAN estimates of incidence and mortality worldwide for 36 cancers in 185 countries. CA Cancer J Clin 68(6):394–424 30207593 10.3322/caac.21492

[2] Fitzmaurice C, Allen C, Barber RM et al (2017) Global, regional, and national cancer incidence, mortality, years of life lost, years lived with disability, and disability-adjusted life-years for 32 cancer groups, 1990 to 2015: a systematic analysis for the Global Burden of Disease Study. JAMA Oncol 3(4):524–548 27918777 10.1001/jamaoncol.2016.5688 PMC6103527

[3] Siegel RL, Miller KD, Jemal A (2018) Cancer statistics. CA Cancer J Clin 68(1):7–30 29313949 10.3322/caac.21442

[4] Chow LQM (2020) Head and Neck Cancer. N Engl J Med 382(1):60–72 31893516 10.1056/NEJMra1715715

[5] Vermorken JB, Specenier P (2010) Optimal treatment for recurrent/metastatic head and neck cancer. Ann Oncol 21(Suppl 7):vii252–vii261 20943624 10.1093/annonc/mdq453

[6] Marur S, Forastiere AA (2008) Head and neck cancer: changing epidemiology, diagnosis, and treatment. Mayo Clin Proc 83(4):489–501 18380996 10.4065/83.4.489

[7] Pisani P, Airoldi M, Allais A et al (2020) Metastatic disease in head & neck oncology. Acta Otorhinolaryngol Ital 40(Suppl. 1):S1-s86 32469009 10.14639/0392-100X-suppl.1-40-2020 PMC7263073

[8] Ferris RL, Blumenschein G, Fayette J et al (2016) Nivolumab for recurrent squamous-cell carcinoma of the head and neck. N Engl J Med 375(19):1856–1867 27718784 10.1056/NEJMoa1602252 PMC5564292

[9] Cohen EEW, Bell RB, Bifulco CB et al (2019) The Society for Immunotherapy of Cancer consensus statement on immunotherapy for the treatment of squamous cell carcinoma of the head and neck (HNSCC). J Immunother Cancer 7(1):184 31307547 10.1186/s40425-019-0662-5 PMC6632213

[10] Yasumatsu R, Shimizu Y, Hanai N et al (2022) Outcomes of long-term nivolumab and subsequent chemotherapy in Japanese patients with head and neck cancer: 2-year follow-up from a multicenter real-world study. Int J Clin Oncol 27(1):95–104 34773525 10.1007/s10147-021-02047-y PMC8732924

[11] Vasiliadou I, Breik O, Baker H et al (2021) Safety and treatment outcomes of nivolumab for the treatment of recurrent or metastatic head and neck squamous cell carcinoma: retrospective multicenter cohort study. Cancers (Basel) 13(6):1413 33808781 10.3390/cancers13061413 PMC8003537

[12] Matsuki T, Okamoto I, Fushimi C et al (2020) Real-world, long-term outcomes of nivolumab therapy for recurrent or metastatic squamous cell carcinoma of the head and neck and impact of the magnitude of best overall response: a retrospective multicenter study of 88 patients. Cancers (Basel) 12(11):3427 33218183 10.3390/cancers12113427 PMC7699139

[13] Wagner SM, Magnes T, Melchardt T et al (2023) Real-world data of palliative first-line checkpoint inhibitor therapy for head and neck cancer. Anticancer Res 43(3):1273–1282 36854497 10.21873/anticanres.16274

[14] Gogate A, Bennett B, Poonja Z et al (2023) Phase 4 multinational multicenter retrospective and prospective real-world study of nivolumab in recurrent and metastatic squamous cell carcinoma of the head and neck. Cancers (Basel) 15(14):3552 37509217 10.3390/cancers15143552 PMC10377225

[15] Kariya S, Shimizu Y, Hanai N et al (2021) Effectiveness of nivolumab affected by prior cetuximab use and neck dissection in Japanese patients with recurrent or metastatic head and neck cancer: results from a retrospective observational study in a real-world setting. Int J Clin Oncol 26(6):1049–1056 33830342 10.1007/s10147-021-01900-4 PMC8134300

[16] Serindere G, Bolgul B, Gursoy D, Hakverdi S, Savas N (2019) Comparison of head and neck cancer distribution in Turkish and Syrian populations. Iran J Public Health 48(10):1810–1816 31850258 PMC6908918

[17] Guo K, Xiao W, Chen X, Zhao Z, Lin Y, Chen G (2021) Epidemiological trends of head and neck cancer: a population-based study. Biomed Res Int 2021:1738932 34337000 10.1155/2021/1738932 PMC8294963

[18] Ferris RL, Blumenschein G, Fayette J et al (2018) Nivolumab vs investigator’s choice in recurrent or metastatic squamous cell carcinoma of the head and neck: 2-year long-term survival update of CheckMate 141 with analyses by tumor PD-L1 expression. Oral Oncol 81:45–51 29884413 10.1016/j.oraloncology.2018.04.008 PMC6563923

[19] Okamoto I, Sato H, Kondo T et al (2019) Efficacy and safety of nivolumab in 100 patients with recurrent or metastatic head and neck cancer - a retrospective multicentre study. Acta Otolaryngol 139(10):918–925 31460818 10.1080/00016489.2019.1648867

[20] Matsuo M, Yasumatsu R, Masuda M et al (2020) Relationship between immune-related adverse events and the long-term outcomes in recurrent/metastatic head and neck squamous cell carcinoma treated with nivolumab. Oral Oncol 101:104525 31863963 10.1016/j.oraloncology.2019.104525

[21] Hori R, Shinohara S, Kojima T et al (2019) Real-world outcomes and prognostic factors in patients receiving nivolumab therapy for recurrent or metastatic head and neck carcinoma. Cancers (Basel) 11(9):1317 31500103 10.3390/cancers11091317 PMC6770631

[22] Otsuki S, Hori R, Shinohara S et al (2022) Real-world 2-year long-term outcomes and prognostic factors in patients receiving nivolumab therapy for recurrent or metastatic squamous cell carcinoma of the head and neck. Auris Nasus Larynx 49(5):834–844 35232636 10.1016/j.anl.2022.02.006

[23] Kim H, Kwon M, Kim B et al (2020) Clinical outcomes of immune checkpoint inhibitors for patients with recurrent or metastatic head and neck cancer: real-world data in Korea. BMC Cancer 20(1):727 32758163 10.1186/s12885-020-07214-4 PMC7405432

[24] Vallianou NG, Evangelopoulos A, Kounatidis D et al (2023) Immunotherapy in head and neck cancer: where do we stand? Curr Oncol Rep 25(8):897–912 37213060 10.1007/s11912-023-01425-1

[25] Hwang M, Canzoniero JV, Rosner S et al (2022) Peripheral blood immune cell dynamics reflect antitumor immune responses and predict clinical response to immunotherapy. J Immunother Cancer 10(6):e004688 35688557 10.1136/jitc-2022-004688 PMC9189831

[26] Valero C, Lee M, Hoen D et al (2021) Pretreatment neutrophil-to-lymphocyte ratio and mutational burden as biomarkers of tumor response to immune checkpoint inhibitors. Nat Commun 12(1):729 33526794 10.1038/s41467-021-20935-9 PMC7851155

[27] Cao D, Xu H, Xu X, Guo T, Ge W (2018) A reliable and feasible way to predict the benefits of Nivolumab in patients with non-small cell lung cancer: a pooled analysis of 14 retrospective studies. Oncoimmunology 7(11):e1507262 30377569 10.1080/2162402X.2018.1507262 PMC6205035

[28] Nakaya A, Kurata T, Yoshioka H et al (2018) Neutrophil-to-lymphocyte ratio as an early marker of outcomes in patients with advanced non-small-cell lung cancer treated with nivolumab. Int J Clin Oncol 23(4):634–640 29442281 10.1007/s10147-018-1250-2 PMC6097082

